# Gender Differences in the Emergence of Post-Traumatic Stress Disorder Following a Single Exposure to a Terrorist Related Crime: A Meta-Analysis

**DOI:** 10.1192/bjo.2024.184

**Published:** 2024-08-01

**Authors:** Huma Riaz Khan, Ahmed Usman Ibrahim

**Affiliations:** Leeds & York Partnership NHS Foundation Trust, Leeds, United Kingdom

## Abstract

**Aims:**

To quantify and evaluate the gender differences regarding the development of PTSD. This meta-analysis calculates (a) the difference between males and females who develop PTSD, and (b) the difference in gendered relative risk of PTSD development.

**Methods:**

Study selection criteria included participant mean age above 18 years, single and direct exposure to a terrorism related traumatic event, and a confirmed diagnosis based on Diagnostic and Statistical Manual of Mental Disorders 5^th^ edition. Data extraction included year and location of terrorist event, the total number of participants in the study, the total numbers of males and females diagnosed with PTSD, and time (in months) of diagnosis following the traumatic event. The number of males and females affected by PTSD was pooled using random effects inverse variance weighted meta-analysis and relative risks (95% confidence interval) were calculated.

**Results:**

Twenty-seven studies met the inclusion criteria of which five had significant information to be included in the meta-analysis. The total number of males in the pooled sample size was 328, and the total number of females was 354 out which a total number of 34 males and 66 females met the PTSD criteria. The mean average of males and females affected by PTSD was 6 and 11, respectively. An independent samples Mann Whitney U test rejected the null hypothesis (*p* < 0.05) and concluded that the distribution of PTSD between males and females was significantly different. The meta-analysis found an overall relative risk of a diagnosis of PTSD in females to be 1.82 (95% CI 1.25–2.65) compared with males.

**Conclusion:**

This meta-analysis found females to have an elevated risk of developing PTSD following a single terrorism traumatic event. The results of our study are supported by previously published research, which has found females to be at higher risks of developing PTSD. However, such research has proposed gender differences secondary to the types of stressful events experienced, which does not apply to our meta-analysis given the uniformity of the traumatic event we explore. Other factors, therefore, need investigating to understand this phenomenon.

We acknowledge that researching psychological consequences in communities affected by terrorism is complicated and limited by lack of healthcare access, trained clinicians, cultural diversity in the expression and articulation of a community's traumatic experience and of course, the instability of the ground fabric. Other limitations of the included studies are the binary of gender reporting, which limits a fuller understanding of a minoritized community.

